# Acetylsalicylic Acid and Head and Neck Cancer: Risk, Five-Year Survival, and Recurrence in over 11,000,000 Patients—A Retrospective Case–Control Study

**DOI:** 10.3390/cancers17132065

**Published:** 2025-06-20

**Authors:** Jonas Wüster, Stefan Heene, Leonard Brandenburg, Robert Preissner, Susanne Nahles, Max Heiland, Saskia Preissner

**Affiliations:** 1 Department of Oral and Maxillofacial Surgery, Medical Center, University of Freiburg, 79085 Freiburg im Breisgau, Germany; stefan.heene@uniklinik-freiburg.de (S.H.); leonard.brandenburg@uniklinik-freiburg.de (L.B.); 2 Institute of Physiology and Science-IT, Charité-Universitätsmedizin Berlin, 10117 Berlin, Germany; robert.preissner@charite.de; 3 Department of Oral and Maxillofacial Surgery, Charité-Universitätsmedizin Berlin, 13353 Berlin, Germany; susanne.nahles@charite.de (S.N.); max.heiland@charite.de (M.H.); saskia.preissner@charite.de (S.P.)

**Keywords:** head and neck cancer, HNC, ASA, aspirin, five-year-survival

## Abstract

**Background/Objectives**: Acetylsalicylic acid (ASA) medication has been suggested to have a beneficial effect on cancer risk and survival. Therefore, this retrospective case–control study investigated the correlation between ASA and HNC in over 11,000,000 patients to investigate the impact of ASA intake on the risk of developing HNC, five-year survival rates, and the likelihood of secondary malignant neoplasms and malignant lymph node involvement. **Methods**: Retrospective clinical data was retrieved from a federated EHR network. Patients prescribed ASA were assigned to Cohort I, while those who were not prescribed ASA were assigned to Cohort II. Moreover, patients diagnosed with HNC, and prescribed ASA were assigned to Cohort III, while those with HNC but no history of ASA use were assigned to Cohort IV. **Results**: After matching, Cohorts I and II included 5,716,056 patients each. HNC incidence was significantly lower (OR 0.88; 95% CI 0.86–0.90) in Cohort I (+ASA) compared to Cohort II (−ASA). Furthermore, five-year survival was higher for patients taking ASA medication (survival probability 67.93%) compared to patients who did not (65.54%). These findings coincide with a lower risk of death of 22.8% (+ASA) compared to 23.6% (−ASA), which was statistically significant (*p* = 0.006). Patients with ASA intake also showed a lower risk of malignant neoplasms of lymph nodes (17.4% vs. 18.5%). **Conclusions**: Our analyses revealed a lower risk of HNC, a higher five-year survival rate, and a lower risk of malignant neoplasms of lymph nodes in patients with ASA medication. However, the retrospective design and the lack of evaluation of confounders limit the significance of our data, and, therefore, further analyses should be considered.

## Simple Summary

Head and neck cancer (HNC) is one of the most common cancers, and has a relatively low survival rate. Acetylsalicylic acid (ASA) is one of the most prescribed drugs and has been shown to have beneficial effects on various types of cancer. Therefore, we investigated the effects of ASA intake on the risk of HNC, five-year survival, and disease recurrence in a retrospective large-cohort case–control study. Our analyses revealed a lower risk of HNC, higher survival rates, and development of fewer malignant lymph nodes in patients taking ASA medication. However, due to the limitations of this study, these findings need to be interpreted cautiously. Nevertheless, this study provides a strong foundation for more detailed research on the effects of ASA in the context of HNC.

## 1. Introduction

Head and neck cancer (HNC), as a heterogeneous group, includes all epithelial malignancies of the oral cavity, pharynx, larynx, nasal cavity, paranasal sinus, and salivary glands (ICD-10 codes C00 to C14). Accounting for more than 660,000 new cases each year, it is the seventh most common cancer globally [1,2,3]. Despite advances in the treatment of HNC—including surgical techniques, radiotherapy, immunotherapy, and chemotherapy—the median five-year survival rate remains unchanged, depending on the localization and tumor stage [4]. Concerning pathogenesis, risk factors such as tobacco and alcohol abuse remain associated with the majority (approximately 75%) of HNC cases [5]. In recent years, however, the incidence of human papillomavirus (HPV)-associated HNC has increased, particularly among younger individuals [6]. Given the high rate of recurrences—especially in HPV-negative and nicotine-dependent patients—there is an urgent need for novel preventive and protective treatment strategies [7].

Recently, the influence of various medications has been further investigated to detect possible links to the risk of the development of HNC and clinical outcomes such as survival [8,9,10]. One of the most often prescribed drugs, which has been sold in tablet form since 1904, is acetylsalicylic acid (ASA, aspirin) [11]. To date, the consumption of this drug amounts to 44,000 tons (approximately 120 billion pills) each year [11,12]. ASA acts via cyclooxygenase (COX/PTGS)-dependent and -independent pathways [13], resulting in different effects on the human body. It is an irreversible COX inhibitor, thus suppressing the production of prostaglandins and thromboxanes. Low-dose use blocks the formation of thromboxane A_2_ in platelets, hence diminishing platelet aggregation. Due to its antithrombotic effect, it has a long-established place in the prevention of cardio- and cerebrovascular diseases [14].

Apart from these effects, studies have also revealed a positive impact on mortality and the development of several types of cancer. In this context, Tsoi et al. found that long-term ASA intake significantly reduces cancer risk [15]. Moreover, ASA medication has been reported to have a protective effect on colorectal cancer in patients with Lynch syndrome [16]. Regarding different types of gastrointestinal cancer, it has been shown that long-term intake is also associated with reduced gastric/stomach cancer incidence and mortality [17,18], while more recent studies confirm the protective effect against esophageal cancer [19]. Furthermore, low-dose ASA medication has likewise been associated with lower risks of pancreatic cancer [20], and hepatocellular carcinoma [21], a lower mortality in lung cancer patients [22], and reduces the overall risk of breast cancer [23].

The potential underlying mechanisms, however, remain poorly understood despite new research conducted in this field. It has been shown that ASA interferes with the expression of many proinflammatory modulators [24,25]; salicylates (SAs), in general, are linked to oxidative cell stress [26]; and SAs and ASA might induce anticancer effects via the activation of stress [27]. Although the mechanisms behind ASA’s anticancer effects are not yet fully understood, there is evidence that it reduces cancer risk and improves five-year survival in several cancer types.

The present study aims to further elucidate the potential association between ASA use and the development and clinical course of HNC. Specifically, this investigation evaluates the impact of ASA on HNC incidence and clinical outcomes, including five-year survival and recurrence rates, in the context of a large case–control study comprising more than 11,000,000 patients. Data for this study were obtained from the TriNetX Global Health Research Network (TriNetX, Cambridge, MA, USA), a real-world data platform. 

## 2. Materials and Methods

### 2.1. Ethics Statement

Following local legislation and institutional requirements, ethical review and approval were not required due to the retrospective nature of this study and the de-identification of all patient data. Similarly, written informed consent was not necessary, as per national legislation and institutional guidelines.

### 2.2. Data Acquisition, Inclusion and Exclusion Criteria, and Patient Matching

The TriNetX Global Health Research Network offers access to electronic medical records from more than 96 healthcare organizations (HCOs) across 30 countries, thereby facilitating the aggregation and exchange of longitudinal clinical data between contract research organizations and pharmaceutical companies. At the time of data retrieval, the database consisted of anonymized health records of over 250 million individuals, which had previously been utilized for large-scale statistical analyses in various studies. For the risk analysis, the TriNetX database was queried for individuals prescribed ASA. These patients were then analyzed for the occurrence of head and neck cancer (HNC) using the International Classification of Diseases, 10th Revision (ICD-10) codes C00-C14. To assess differences in survival and the occurrence of secondary malignant neoplasms and malignant lymph nodes, the database was queried for patients diagnosed with HNC. Eligible patients were those diagnosed with HNC between five and twenty years prior to the access date (6 February 2025) and with at least five years (1825 days) of documented follow-up after their initial inpatient encounter. Patients were excluded if their HNC was diagnosed more than 20 years ago.

**Figure 1:**
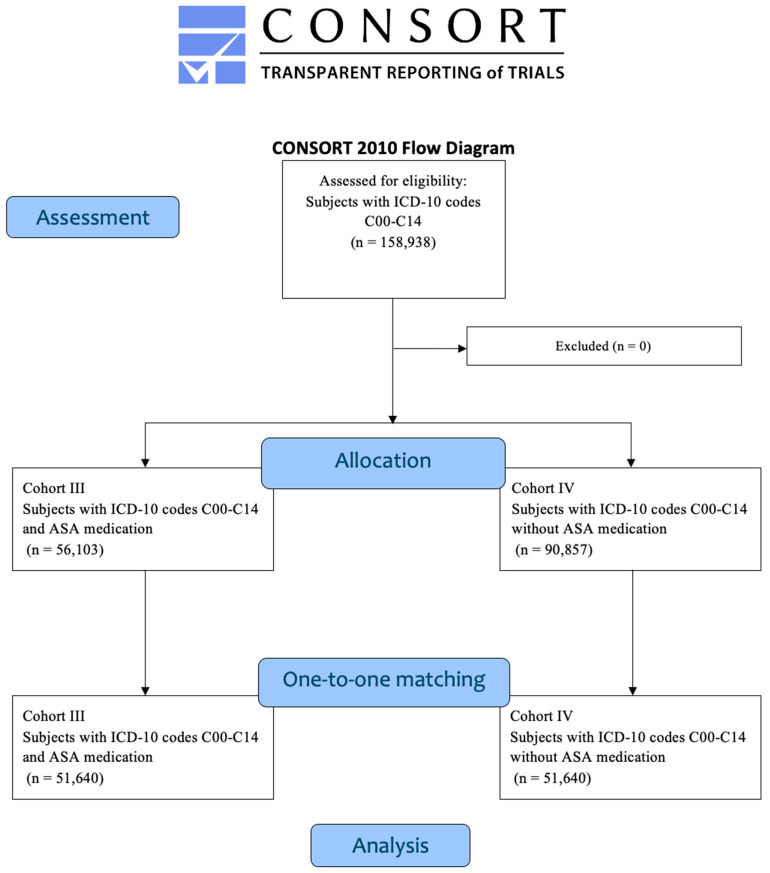
CONSORT flowchart for Cohorts III and IV.

Afterwards distinct analyses were conducted. First, patients taking ASA medication were assigned to Cohort I, while those without a history of ASA use formed Cohort II. Propensity score matching was performed based on age and gender to ensure comparability, and the incidence of HNC (ICD-10 codes C00–C14) was analyzed in both cohorts. In a second analysis, patients who were diagnosed with HNC (ICD-10 codes C00-C14) and used ASA were assigned to Cohort III, while those with HNC but no history of ASA use were placed in Cohort IV (Figure 1). The cohorts were evaluated for overall survival and the development of secondary malignancies, including secondary malignant neoplasms of the respiratory and digestive organs (ICD-10 code C78), secondary malignant neoplasms of other and unspecified sites (ICD-10 code C79), malignant neoplasms of unspecified sites (ICD-10 code C80), and secondary and unspecified malignant neoplasm of lymph nodes (ICD-10 code C77). To minimize confounding and ensure balanced covariate distributions, one-to-one propensity score matching was performed based on age, gender, and history of tobacco and/or alcohol abuse (ICD-10 codes F10.1, F10.2, F17), as previously described [28].

### 2.3. Data Analysis

The primary outcome of interest was all-cause mortality, with survival analyses conducted using Kaplan–Meier estimates. Cox proportional hazard regression was employed to calculate hazard ratios (HRs), while risk ratios (RRs) and odds ratios (ORs) were determined for each cohort. Statistical analyses were restricted to a five-year follow-up period following the initial HNC diagnosis, as patients without disease recurrence or metastasis within this timeframe were considered disease-free. The log-rank test was applied to compare survival distributions, with statistical significance set at *p* < 0.05.

## 3. Results

### 3.1. Assessment, Allocation, and Matching of Cohorts I and II

After matching, Cohorts I and II each consisted of 5,716,056 patients, with a mean age [±SD] at tumor diagnosis of 62.5 ± 17.5 years. Due to their outcomes prior to the time window, 39,295 patients in Cohort I and 34,477 patients in Cohort II had to be excluded. The patient characteristics of all cohorts before and after matching are visualized in Table 1.

### 3.2. Risk Analyses for ASA Medication and HNC Diagnosis

**Figure 2:**
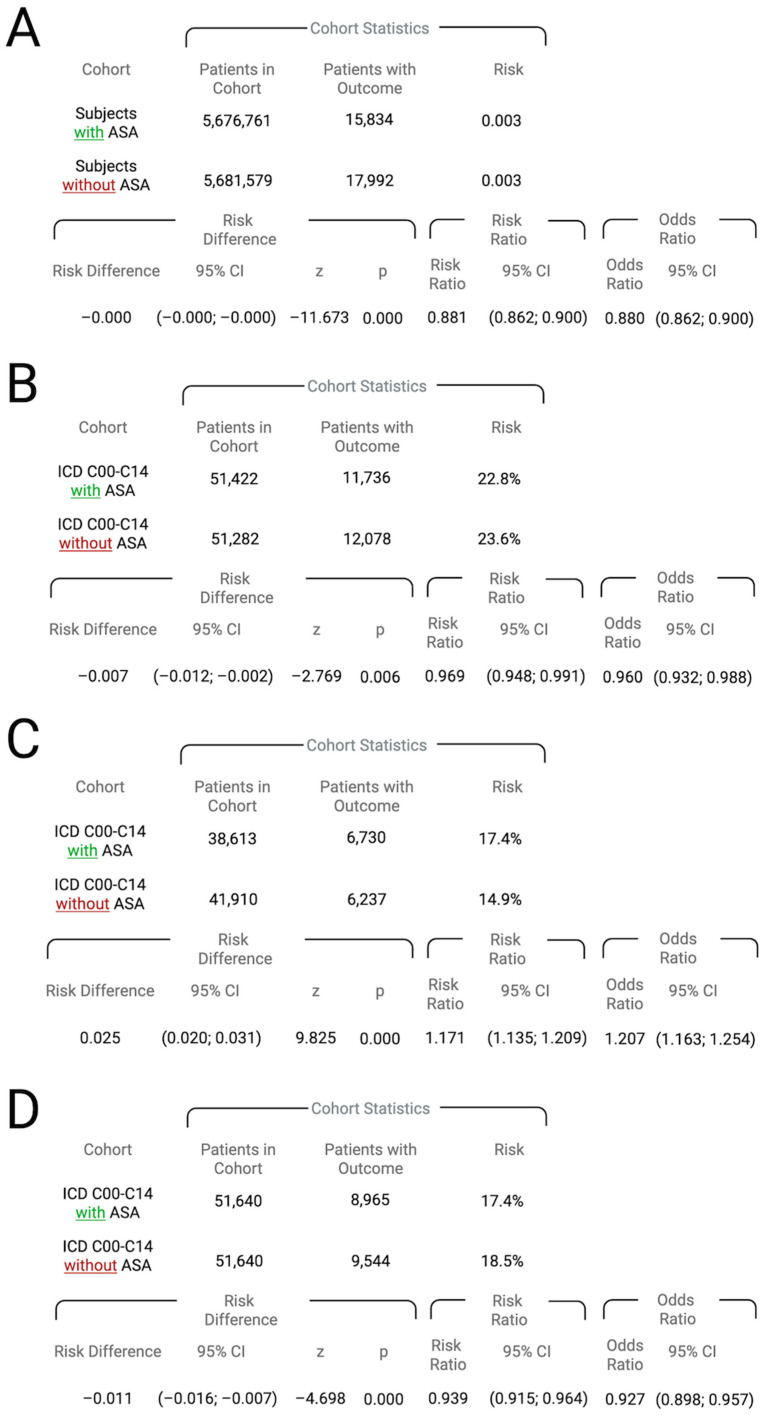
(**A**) Comparison of Cohort I (patients prescribed ASA) and Cohort II (patients not prescribed ASA) in terms of HNC (ICD-10 codes C00–C14) diagnosis risk, including risk ratios and odds ratios. (**B**) Risk of death in Cohort III (ICD-10 codes C00–C14 with ASA use) versus Cohort IV (ICD-10 codes C00–C14 without ASA use), including risk ratios and odds ratios. (**C**) Incidence of secondary malignant neoplasms in Cohorts III and IV, presented with corresponding risk ratios and odds ratios. (**D**) Incidence of malignant lymph node involvement in Cohorts III and IV, including associated risk ratios and odds ratios.

First, statistical analyses regarding ASA medication and HNC diagnosis were performed. HNC incidence was slightly lower in Cohort I compared to Cohort II, with a risk ratio of 0.881 (95% CI: 0.862–0.900) and an odds ratio of 0.880 (95% CI: 0.862–0.900), showing a statistically significant, but still minimal, risk difference (*p* < 0.001) (Figure 2A).

### 3.3. Assessment, Allocation, and Matching of Cohorts III and IV

After matching, Cohorts III and IV each consisted of 51,640 patients, with a mean age of 66.3 ± 12.2 years in Cohort III and 66.6 ± 12.5 years in Cohort IV. The patient characteristics are listed in Table 2.

### 3.4. Risk Analysis for HNC-Diagnosed Patients and ASA Regarding Overall Survival

**Figure 3:**
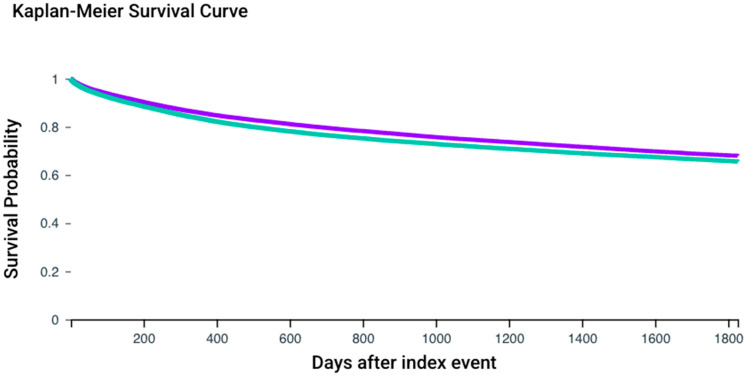
Kaplan–Meier survival curves for both cohorts. Cohort III (ICD-10 codes C00–C14 and ASA medication; purple) and Cohort IV (ICD-10 codes C00–C14 without ASA medication; green).

To further analyze the influence of ASA medication in HNC-diagnosed patients, Cohorts III and IV were compared. A total of 218 patients from Cohort III and 358 patients from Cohort IV were excluded due to the occurrence of the outcomes prior to the defined observation window. During the period following the initial diagnosis of HNC, 11,736 patients in Cohort III and 12,078 patients in Cohort IV died (Figure 3), corresponding to a risk of death of 22.8% and 23.6%, respectively (Figure 2B). The related risk ratio (RR) was 0.969 (95% CI: 0.948–0.991), and the odds ratio (OR) was 0.960 (95% CI: 0.932–0.988). The survival probability was 67.93% for Cohort III and 65.54% for Cohort IV (log-rank test: χ^2^ = 86.505, *p* < 0.001), and the hazard ratio was 0.886 (95% CI: 0.864–0.909; *p* < 0.001) (Figure 2B).

### 3.5. Risk Analysis of HNC-Diagnosed Patients and ASA Regarding Secondary Malignant Neoplasm

Furthermore, an investigation of the risk of secondary malignant neoplasm was conducted for both cohorts. In total, 13,027 patients from Cohort III and 9730 patients from Cohort IV had to be excluded due to outcomes prior to the time window. Of the remaining patients in Cohort III (38,613 patients), 6730 patients developed a secondary malignancy, corresponding to a risk of 17.4%. In Cohort IV (41,910 patients), 6237 patients developed a secondary malignancy, corresponding to a risk of 14.9%. The absolute risk difference was 0.025 (95% CI: 0.020–0.031; *p* < 0.001), the risk ratio was 1.171 (95% CI: 1.135–1.209), and the odds ratio was 1.207 (95% CI: 1.163–1.254) (Figure 2C).

### 3.6. Risk Analysis for HNC-Diagnosed Patients and the Development of Malignant Lymph Nodes

Risk analysis was conducted to compare secondary and unspecified malignant neoplasms of lymph nodes in Cohorts III and IV. In Cohort III, 8965 patients developed a secondary and unspecified cancer of the lymph nodes, which corresponds to a risk of 17.4%. In Cohort IV, 9544 patients were affected, with a corresponding risk of 18.5%. The absolute risk difference was −0.011 (95% CI: −0.016 to −0.007; *p* < 0.001), the risk ratio was 0.939 (95% CI: 0.915–0.964), and the odds ratio was 0.927 (95% CI: 0.898–0.957) (Figure 2D).

## 4. Discussion

This study investigated the influence of ASA medication on the development of HNC and the clinical outcomes by analyzing five-year survival and the risk of secondary malignant neoplasms and malignant lymph nodes. To the best of the authors’ knowledge, this is the first study to address this topic using a cohort of such magnitude. The findings suggest a potentially beneficial effect of ASA use, both in reducing the risk of developing HNC and in improving survival outcomes among HNC patients, compared to those not receiving ASA therapy. Moreover, patients diagnosed with HNC and ASA medication showed a lower risk of the development of malignant lymph nodes.

Recently, due to the discovery of its beneficial effects on various types of cancer, there has been a growing interest in ASA. In this context, Tsoi et al. found that long-term aspirin intake significantly reduces cancer risk in general [15]. Specifically, ASA medication has been reported to reduce the risk of colorectal cancer in patients with Lynch syndrome [16], stomach cancer [17,18], esophageal cancer [19], pancreatic cancer [20], hepatocellular carcinoma [21], and lung cancer [22]. Therefore, ASA has gained increasing attention, as traditional risk factors, such as tobacco and alcohol abuse, are linked to HNC and vascular disease. Regarding vascular disease, the American College of Cardiology (ACC) and the American Heart Association (AHA) recommend medication like aspirin, statins, angiotensin-converting enzyme (ACE) inhibitors, and smoking abstinence, as each of these interventions might reduce major adverse cardiovascular events in patients [29,30,31]. Chen et al. demonstrated that most patients with vascular disease (84%) receive ASA medication [32]. Accordingly, we assumed that there are many patients at risk of HNC who are regularly taking ASA.

However, regarding HNC, evidence on the effects of non-steroidal anti-inflammatory drugs (NSAIDs) and ASA in particular is inconclusive. In 2006, a first connection between ASA intake and HNC was established, indicating a lower risk of HNC development [33]. Subsequently, several investigations confirmed these findings for ASA but did not find similar results for other NSAIDs [34,35]. More recently, the survival and cancer outcome of HNC patients receiving ASA medication was analyzed, but no significant effects were found for HNC as a heterogeneous group [36,37]. Certain subgroups, however, like patients with advanced-stage OSCC or HPV-positive OPSCC, were shown to have an increased possibility of survival due to ASA intake [37,38]. Additionally, a 2021 systematic review also revealed that NSAIDs contributed to lower cancer-specific mortality overall, but it did not indicate a protective effect of ASA in particular [39]. Further investigations reveal a lower risk of disease recurrence in HNC patients [39] and OPSCC patients [36] with ASA medication. As the mentioned studies analyze populations up to a maximum of only 10,000 patients, we aimed to investigate the effects of aspirin on HNC in a larger cohort, addressing the risk of HNC, the five-year survival rate, and the risk of secondary malignant neoplasm and malignant lymph nodes.

Despite the described effects of ASA medication on HNC, the underlying mechanisms remain unclear. Zhang et al. found that ASA suppresses the activation of focal adhesion kinase (FAK) and the phosphorylation of Akt, NF-κB, and STAT3; it was also found to downregulate OSCC cell proliferation, colony formation, invasion, and migration and upregulate apoptosis in OSCC cell lines [40]. Further investigations found that ASA induces apoptosis in OSCC cells via the activation of caspases, the downregulation of Mcl-1, and the inactivation of ERK-1/2 and AKT [41]. Yang et al. discovered that COX-2 selective inhibitors (which do not include ASA) might play an important role in the generation of biochemical mediators that stimulate the growth of human oral cancer cell lines [42]. In general, COX-1 is constitutively expressed in most tissues, whereas COX-2 is induced in association with pathological inflammatory sites such as human cancer [43,44,45]. Additionally, COX-2 can be found in the tumor microvasculature of many human tumors [46,47], directly impacting tumor growth through angiogenic endothelial cell growth and blood vessel formation via COX-2-derived prostaglandin E_2_ (PGE_2_) [48].

Nevertheless, the results must be interpreted with caution due to several limitations. In particular, patient identification relied on diagnostic coding (ICD-10 codes C00–C14), which assumes accurate classification of malignant neoplasms in the head and neck region. Furthermore, this study may have included individuals with various subtypes or rare entities of HNC, potentially influencing the generalizability of the findings. Moreover, all ASA medication was regarded as one group despite possible dose-related effects [49]. Information on the duration of ASA therapy was also not available, but, in general, ASA is administered on a long-term basis. Although clinical, histological, and molecular characteristics—as well as disease staging and therapeutic regimens—are well-established determinants of survival in patients with HNC, these variables were not accounted for in the present analysis [50,51,52]. Despite the implementation of one-to-one propensity score matching, the possibility of residual confounding remains, primarily due to the lack of detailed information on tobacco use (e.g., pack years) [53], alcohol consumption (e.g., units per week), racial background [54], comorbidities [55,56], and HPV status [57]. For example, a review demonstrated that nine out of twelve studies indicated a dose–response relationship between cigarette smoking and the risk of HNC [53]. In the context of the present study, it was only possible to record whether a diagnosis of nicotine dependence was present (ICD-10 code F17), and there was no information on the duration or extent of nicotine and/or other substance use. The same limitation applies to alcohol consumption among the included patients. Based solely on the recorded ICD codes, no conclusions can be drawn regarding the quantity or frequency of alcohol intake. However, the literature describes an increased risk of HNC in relation to the amount of alcohol consumed [58,59]. Furthermore, higher alcohol consumption has been associated with an increased risk of cancer recurrence, particularly at a level of 8–14 drinks per week [60]. Due to the retrospective nature of the current study, such dose-dependent associations cannot be fully captured, which may limit the interpretability and strength of the study findings. In addition, other systematic biases need to be considered, as patients in the ASA cohort might be more adherent to healthcare in general.

Nonetheless, the large cohort size, exceeding 5,000,000 patients per group in the HNC risk analysis, combined with the matching procedure, likely contributed to a partial mitigation of these differences. Furthermore, the quality of the data extracted from the TriNetX database is considered high, as it adheres to the rigorous standards set by the National COVID Cohort Collaborative (N3C). The observed association between aspirin use and a reduced risk of developing HNC, improved five-year survival, and a lower incidence of secondary malignancies may prompt further investigations into this potential protective effect. Should these findings be corroborated by future studies, aspirin could represent a valuable addition to preventive strategies in HNC management.

## 5. Conclusions

Aspirin use was found to correlate with a lower risk of HNC and a higher survival rate after five years (67.93%) compared to patients not taking such medication (65.54%). Furthermore, patients with ASA treatment showed a lower risk of malignant neoplasms of lymph nodes following HNC diagnosis (17.4% versus 18.5%). Further research is required to confirm these findings and unravel the underlying mechanisms, which may lead to new supplementary treatment options in HNC therapy.

## Figures and Tables

**Table 1:** Characteristics of Cohort I and Cohort II before and after matching for age and gender.

	Before Matching				After Matching			
Patients (n)	Cohort I	Cohort II	*p*-Value	Standardized Mean Difference	Cohort I	Cohort II	*p*-Value	Standardized Mean Difference
Total	5,716,056	16,691,409	<0.001	1.012	5,716,056	5,716,056	1	<0.001
Female	2,874,632 (50.3%)	9,637,464 (57.7%)	-	-	2,874,632 (50.3%)	2,874,632 (50.3%)	-	-
Male	2,841,424 (49.7%)	7,053,945 (42.3%)	-	-	2,841,424 (49.7%)	2,841,424 (49.7%)	-	-
Mean age at diagnosis (years)	62.5	41.0		-	62.5	62.5		
Standard deviation	17.5	24.5		-	17.5	17.5		

**Table 2:** Characteristics of Cohort III and Cohort IV before and after matching for age, gender, tobacco use, and alcohol abuse.

	Before Matching				After Matching			
Patients (n)	Cohort III	Cohort IV	*p*-Value	Standardized Mean Difference	Cohort III	Cohort IV	*p*-Value	Standardized Mean Difference
Total	56,103	90,857	<0.001	0.521	51,640	51,640	<0.001	0.029
Female	17,779 (31.7%)	29,948 (33.0%)	-	-	16,595 (32.1%)	17.078 (33.1%)	-	-
Male	38,324 (68.3%)	60,909 (67.0%)	-	-	35,045 (67.9%)	34,562 (66.9%)	-	-
Mean age at diagnosis (years)	66.7	59.2		-	66.3	66.6		
Standard deviation	12.1	16.4		-	12.2	12.5		
ICD-10 F17	14,144 (25.2%)	10,193 (11.2%)	<0.001	0.369	10,290 (19.9%)	9716 (18.8%)	<0.001	0.028
ICD-10 F10.1	4594 (8.2%)	2879 (3.2%)	<0.001	0.218	2973 (5.8%)	2698 (5.2%)	<0.001	0.023
ICD-10 F10.2	3482 (6.2%)	2423 (2.7%)	<0.001	0.173	2338 (4.5%)	2191 (4.5%)	0.025	0.014

## Data Availability

Data can be retrieved from the TriNetX network (https://trinetx.com). Public access to the database is closed, but a request can be made to TriNetX (https://live.trinetx.com). The datasets are available from the corresponding author upon reasonable request.
